# Perspectives on the impact of painful diabetic peripheral neuropathy in a multicultural population

**DOI:** 10.1186/s40842-017-0051-2

**Published:** 2017-12-28

**Authors:** Martin Eichholz, Andrea H. Alexander, Joseph C. Cappelleri, Patrick Hlavacek, Bruce Parsons, Alesia Sadosky, Michael M. Tuchman

**Affiliations:** 1 Kelton Communications, Culver City, CA USA; 2 Pfizer Inc., 235 East 42nd Street, New York, NY 10017 USA; 3 Pfizer Inc., Groton, CT USA; 4 Palm Beach Neurological Center, Palm Beach Gardens, FL USA

**Keywords:** Painful diabetic peripheral neuropathy, Race, Ethnicity, Pain, Productivity

## Abstract

**Background:**

Since few studies have characterized painful diabetic peripheral neuropathy (pDPN) symptoms in multicultural populations, this study fielded a survey to better understand pDPN and its impact in African-American, Caucasian, and Hispanic populations.

**Methods:**

Kelton fielded a survey by phone or Internet, in English or Spanish, among adults with pDPN symptoms in the United States between August and October 2015; African-Americans and Hispanics were oversampled to achieve at least 500 subjects for each group. Patients were required to have been diagnosed with pDPN or score ≥ 3 on ID Pain validated screening tool. The survey elicited information on pDPN symptoms and interactions with healthcare providers (HCPs), and included the Brief Pain Inventory and pain-specific Work Productivity and Assessment Questionnaire (WPAI:SHP).

**Results:**

Respondents included 823 Caucasians, 525 African-Americans, and 537 Hispanics; approximately half of African-Americans and Hispanics were <40 years of age, vs 12% of Caucasians. Pain was less likely to be rated moderate or severe by African-Americans (65%) and Hispanics (49%) relative to Caucasians (87%; *p* < 0.05). African-Americans and Hispanics were less likely than Caucasians to report experiencing specific pDPN sensory symptoms. Significantly fewer African-Americans and Hispanics reported receiving a pDPN diagnosis relative to Caucasians (*p* < 0.05), and higher proportions of African-Americans and Hispanics reported difficulty communicating with their HCP (*p* < 0.05). WPAI:SHP activity impairment was lower in Hispanics (43%) relative to African-Americans (53%) and Caucasian (56%; *p* < 0.05).

**Conclusions:**

Multicultural patients reported differences in pDPN symptoms and pain relative to Caucasians, and fewer received a pDPN diagnosis. While further evaluation is needed to understand these differences, these data suggest a need to broaden pDPN educational initiatives to improve patient-HCP dialogue and encourage discussion of pDPN symptoms and their impact in a multicultural setting.

**Electronic supplementary material:**

The online version of this article (10.1186/s40842-017-0051-2) contains supplementary material, which is available to authorized users.

## Background

Diabetic peripheral neuropathy (DPN) is a common complication of Type 1 and Type 2 diabetes that is characterized by nerve damage. When DPN presents with painful symptoms the condition is known as painful diabetic peripheral neuropathy (pDPN). While the epidemiology of pDPN has not been well-characterized, an overall prevalence of 15% has been estimated in the diabetic population [1]. However, prevalence rates exceeding 30% in patients with diabetes have been reported in more recent regional studies [2, 3], and a systematic review of neuropathic pain in the general population reported a pDPN prevalence of 0.8% that represents approximately 26% of individuals with Type 2 diabetes [4].

The substantial patient and economic burdens associated with pDPN are well-recognized and include reductions in patient function, quality of life, and productivity [5, 6], as well as greater healthcare resource utilization and costs relative to patients with diabetes and with DPN without pain [7].

Despite studies evaluating quality of life and other patient-reported outcomes in pDPN, there are limited data on the severity and impact of painful pDPN symptoms from the patient’s perspective. A survey in patients and clinicians who treat patients with diabetes not only showed that misperceptions on the cause and management of pDPN were common in both stakeholder groups but also indicated additional disparities between patient and clinician perspectives regarding communication, severity, and treatment [8]. However, less is known about the patient perceptions of pDPN and interactions between these patients and their healthcare providers (HCPs) in a multicultural population. Therefore, the objective of this study was to characterize the impact of pDPN and identify barriers to its management in a multicultural US population with a focus on African-Americans and Hispanics relative to Caucasians.

## Methods

### Design and populations

Kelton fielded a survey among pDPN patients in the United States between August and October 2015. For inclusion, patients were required to be adults (≥ 18 years old) who self-reported being diagnosed with either Type 1 or Type 2 diabetes *and* either self-reported having received a diagnosis of pDPN by an HCP *or* had a score ≥ 3 on ID Pain [9] (i.e., experienced ≥3 of the following symptoms within the past week: pins and needles, hot/burning, numbness, electrical shocks, or pain that is made worse with the touch of clothing or bed sheets). ID Pain is a validated measure that is used to screen patients for the presence of neuropathic pain based on its demonstrated ability to discriminate between nociceptive and neuropathic pain [9].

The survey, which was developed without patient input but in collaboration with experts in the field, including clinicians, was administered by Internet among Caucasians, and by either Internet or phone among African-Americans and Hispanics, with Internet respondents recruited from a national research panel and phone respondents recruited from purchased phone lists. Oversampling via phone was performed to achieve a goal of at least 500 Hispanic patients and 500 African-American patients. The survey could be completed in English or Spanish, with the Spanish version back-translated by native Spanish-speakers to ensure accuracy of the questionnaire.

The survey (Additional file 1) consisted of batteries of questions that were in part derived from a previous, similar survey [8]. The goal was to capture perspectives on pDPN symptoms (numbness; pins and needles; pain or discomfort at night; tingling or prickling sensation; sensitivity to touch; burning pain or sensation; shooting pain; radiating pain; stinging; stabbing pain; electric shock-like symptoms or sudden pain attacks; throbbing pain), perceptions of pain associated with the symptoms, and how patients discuss these symptoms with their physician.

Additionally, the survey included the Brief Pain Inventory (BPI) [10] and the Work Productivity and Assessment Questionnaire disease-specific version (WPAI:SHP) adapted for pain [11], both of which demonstrate sound psychometric measurement properties and have been used as outcomes across a wide variety of disease states. The BPI rates worst, least, and average pain in the past 24 h and the average pain subscale was used to categorize pain as mild, moderate, and severe based on established cut points for the average pain scale of 0–3 for mild, 4–6 for moderate, and 7–10 for severe [12]. The WPAI:SHP measures impact of the disease on productivity at work due to absenteeism (work time missed), presenteeism (impairment while at work), overall work impairment, and activity impairment outside of work during the past 7 days.

### Statistical analysis

Survey results reflect an unweighted sample. The margin of error was ±3.1% for the total patient sample and 4.0% for the oversampled groups. Analyses for categorical data and continuous data were conducted using chi-square tests and *t*-tests, respectively. The impact of ethnicity was explored based on the combined main sample and oversample and controlled for effects of age, education, and household income using layered cross-tabulations (chi-square tests) and stepwise linear regression [13]. The cross-tabulations were conducted using 16 demographic strata: 3 age groups (18–34 years, 35–54 years, and ≥55 years), 6 education levels, and 7 income levels shown in the demographics table (Table 1).

**Table 1:** Demographic characteristics of the patient populations

Variable	Value		
	Caucasians (*n* = 823)	African-Americans (*n* = 525)	Hispanics (*n* = 537)
Sex, %			
Male	43	48	42
Female	57	52	58
Age, years, mean	55.7^ab^	41.0	37.0
Age distribution, %			
18–29 years	3^ab^	25^b^	21
30–39 years	9^ab^	24	38
40–49 years	16^b^	20	24
50–59 year	30^ab^	18^b^	12
60–69 years	30^ab^	10^b^	6
≥ 70 years	12^ab^	3^b^	1
Marital status, %			
Married or living as married	57^ab^	45^b^	72
Living with domestic partner	4^ab^	11	8
Single, never married	14^ab^	30	16
Widowed	5^b^	4^b^	2
Separated	2^b^	3^b^	1
Divorced	18^ab^	7^b^	2
Education, %			
Less than high school	4^b^	6	7
High school	22^b^	25	42^a^
Some college—no degree	31^b^	28	20^a^
Associate’s degree	16^b^	15	9^a^
Bachelor’s degree	17	18	19
Post-graduate degree	10^b^	8	2^a^
Employment status, %			
Employed	38^ab^	65	69
Retired	31^ab^	12^b^	4
Disabled	19^ab^	10^b^	2
Stay-at-home parent/spouse	9^ab^	5^b^	15
Unemployed, looking for work	2^ab^	4	5
Unemployed, not looking for work	2^b^	2	4
Full time student	< 1^b^	1	2
Annual income, mean	$52,300^b^	$53,700^b^	$58,500
Insurance, %			
Medicare	44^ab^	16^b^	8
Medicaid	14^b^	18	20
Private	33^ab^	47	52
Other	6^ab^	3	2
No insurance	4^ab^	15	18

^a^ *p* < 0.05 vs African-Americans

^b^ *p* < 0.05 vs Hispanics

Stepwise linear regression was also performed among the main sample, using pain severity as the dependent variable and 10 items related to the patients’ experience with symptoms as independent variables (numbness; pins and needles; pain or discomfort at night; tingling or prickling sensation; sensitivity to touch; burning pain or sensation; shooting pain; stinging; stabbing pain; electric shock-like symptoms or sudden pain attacks). All analyses were performed using IBM® SPSS® Statistics 23.

## Results

### Respondent populations

Table 1 presents the demographic characteristics of the multicultural populations, and shows that mean age was significantly higher (*p* < 0.05) among Caucasians than African-Americans and Hispanics, and differences were also observed in the age distribution. Almost half of the African-Americans (49%) and more than half of the Hispanics (59%) were under 40 years of age, compared with only 12% of Caucasians. Caucasians had the lowest rate of employment and the highest rate of retirees among the three cultural groups, and annual income was highest in Hispanics, lowest among Caucasians. Consistent with the older demographic, a significantly greater proportion of Caucasians relative to the other groups had health insurance through Medicare, and a significantly lower proportion were uninsured (both *p* < 0.05) (Table 1); private insurance was the primary insurance type among both African-Americans and Hispanics.

While mean time since diabetes diagnosis was slightly but significantly higher among Caucasians (10.9 years) relative to African-Americans (9.4 years) and Hispanics (9.4 years) (both *p* < 0.05), the medians were similar across ethnicities, 8 years, 8 years, and 9 years, respectively.

### Pain and sensory symptoms

**Fig. 1:**
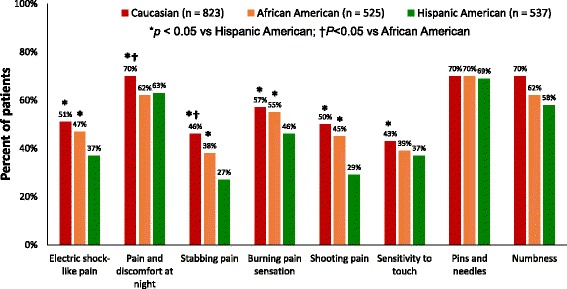
Type of symptoms

African-American and Hispanic patients were less likely than Caucasians to experience a range of sensory symptoms (Fig. 1) that are characteristic of neuropathic pain including some symptoms that appear to drive pain severity such as sensitivity to touch and shooting pain. The layered cross-tabulations of the six symptoms that were significant by ethnicity (electric shock-like pain; pain and discomfort at night; stabbing pain; burning pain sensation; shooting pain; sensitivity to touch) show that these differences by ethnicity generally hold for stabbing pain, with a significant effect of ethnicity for 12 of the 16 strata (*p* < 0.05); shooting pain, which was significant for 11 strata *p* < 0.05); and electric shock pain (*p* < 0.05: for 9 strata) (Table 2). However, significant differences (*p* < 0.05) by ethnicity were limited for pain and discomfort at night (only 4 strata showed a significant effect of ethnicity), and burning pain and sensitivity to touch (each with 6 strata that showed an ethnicity effect).

**Table 2:** Layered cross-tabulation for the effect of ethnicity on the percent of respondents who currently experience the pain symptoms that were significant by ethnicity

Strata	*p*-value					
	Stabbing pain	Shooting pain	Electric shock-like pain	Pain and discomfort at night	Burning pain	Sensitivity to touch
Controlling for age						
18–34 years	< 0.0001	< 0.0001	0.002	NS	0.001	0.004
35–54 years	< 0.0001	< 0.0001	< 0.0001	< 0.001	< 0.0001	0.001
≥ 55 years	NS	NS	NS	NS	NS	NS
Controlling for education						
Less than high school	0.002	0.034	NS	0.021	NS	NS
High school	< 0.0001	< 0.0001	0.006	< 0.0001	<0.0001	NS
Some college – no degree	< 0.0001	0.003	0.007	NS	NS	0.004
Associate’s degree	NS	0.024	NS	NS	NS	NS
Bachelor’s degree	NS	NS	NS	NS	NS	NS
Post-graduate degree	0.009	0.031	NS	NS	0.024	0.007
Controlling for income						
< $25,000	0.006	NS	NS	NS	NS	NS
$25,000 - $34,999	< 0.0001	< 0.0001	0.002	NS	NS	0.004
$35,000 - $49,999	< 0.0001	< 0.0001	0.009	0.005	0.006	NS
$50,000 - $74,999	0.006	0.014	0.018	NS	NS	NS
$75,000 - $99,999	NS	0.001	0.013	NS	NS	NS
$100,000 - $149,999	0.004	NS	0.028	NS	0.001	0.036
≥ $150,000	0.007	NS	NS	NS	NS	NS

*Abbreviations*: *NS* not significant

A stepwise regression analysis with average pain severity in the past year as dependent variable and the 10 pain symptoms as independent variables showed that sensitivity to touch is the strongest predictor of pain, being responsible for 20% of the total *explained* variance in overall pain scores. The second strongest predictor was shooting pain (17%), followed by electric shock-like pain (10%). The overall model was significant (*p* < 0.05), with R^2^ = 0.29 and F = 59.077.

**Fig. 2:**
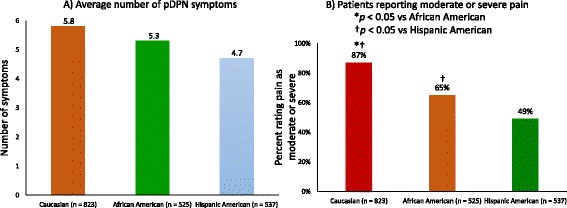
Presence of pDPN symptoms. **a**) Average number of symptoms. **b**) Presence of moderate or severe pain

While the average number of reported pDPN symptoms was lower among African-Americans (5.3) and Hispanics (4.7) relative to Caucasians (5.8), the differences were not significant (Fig. 2a). However, African-Americans and Hispanics were less likely to rate their pain as moderate or severe, 65% and 49%, respectively, relative to Caucasians (87%; both *p* < 0.05) (Fig. 2b). This finding was confirmed through a stepwise linear regression where ethnicity (operationalized as 3 dummy variables, one each for Caucasian, African-American, and Hispanic) as well as age, education, and household income were used as independent variables to predict reported pain levels. The results of the overall significant model show that being Hispanic is the strongest significant predictor of the experienced pain levels (standardized beta coefficient of −0.297), followed by education (beta of 0.211) and being African-American (beta of −0.125). No other independent variable added significant explanatory power.

### Patient and healthcare provider dialogue

**Fig. 3:**
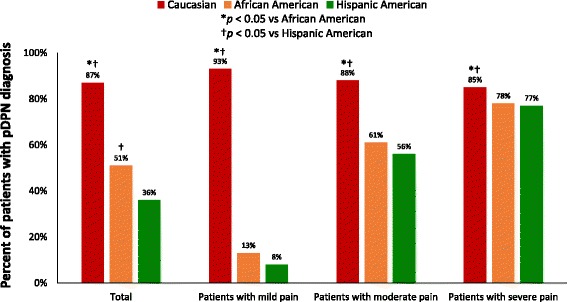
Patients who received a diagnosis of painful diabetic peripheral neuropathy

The proportion of Caucasians who reported receiving a diagnosis of pDPN (87%) was significantly higher than that of African-Americans (51%) and Hispanics (36%) (all *p* < 0.05) (Fig. 3). This significance based on ethnicity was retained in layered cross-tabulations, with 13 of the 16 strata showing significance (*p* ≤ 0.001; only post-graduate degree and income levels of $100,000–$149,999 and ≥$150,000 were not significant). Similar patterns were observed when stratified by pain severity; consistently and significantly higher proportions of Caucasians reported a pDPN diagnosis relative to the other two populations across severity levels (all *p* < 0.05), and Hispanics generally reported the lowest rate of diagnosis, although the differences were not significant vs African-Americans.

**Fig. 4:**
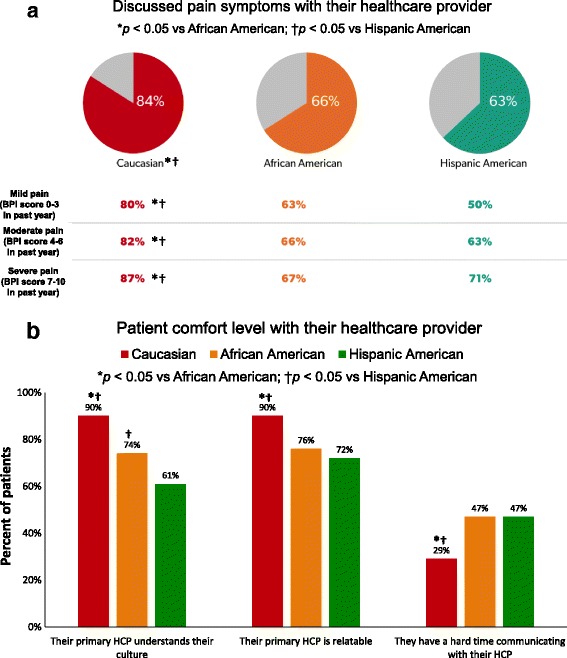
Interaction between patients and their healthcare providers. **a**) Discussion of pain. **b**) Comfort level of patients with their healthcare providers

Significantly lower proportions of African-American and Hispanic patients relative to Caucasians reported discussing their pain symptoms with their healthcare provider across pain severity levels, (all *p* < 0.05) (Fig. 4a). Additionally, among both the African-American and Hispanic populations, there was consistently less comfort with their healthcare providers (Fig. 4b), as indicated by significantly lower proportions of African-Americans and Hispanics who reported that they thought their HCP understood their culture, as well as a harder time communicating.

### Impact of pDPN

**Fig. 5:**
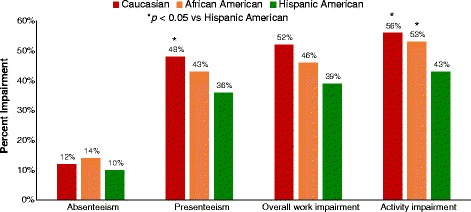
Work loss and productivity impairment assessed using the WPAI:SHP

Overall work impairment due to pain was substantial among employed patients in the three populations (Fig. 5). While Caucasians reported greater work impairment than African-Americans and Hispanics, none of the differences between groups was significant. Presenteeism was at least three times as high as absenteeism in all populations, and presenteeism among Caucasians was significantly higher relative to Hispanics, 48% and 36%, respectively (*p* < 0.05). Activity impairment was significantly (*p* < 0.05) higher among Caucasians (56%) relative to African-Americans (53%) and Hispanics (43%) (Fig. 5).

## Discussion

This study suggests not only that there are significant disparities across cultural groups in their interaction with HCPs regarding pDPN and its symptoms, but that presentation of pDPN itself is also significantly different across these groups, with lower pain severity and fewer number of pDPN symptoms reported among African-Americans and Hispanics relative to Caucasians. In particular, among the types of symptoms, only for pins and needles was there concordance among all three cultural groups for the percentage of patients reporting this symptom. For the other symptoms, the percent of patients reporting the symptoms was generally lowest among Hispanics and highest among Caucasians.

While it has previously been reported that there are differences in how ethnic groups perceive and report types and severity of experimental pain [14, 15], which may in part result from genetic as well as cultural factors [16, 17], the observations here contrast with a recent review indicating that Hispanics report greater pain sensitivity and experience greater severity relative to non-Hispanic Whites [15]. However, it is also possible that these perceptions may be dependent on the type of pain, i.e., neuropathic or nociceptive. Whether these differences extend to the clinical setting has not been adequately explored, although the results reported here do suggest potential differences as well as the need for further evaluating pain perceptions in multicultural populations, including sensations related to neuropathic pain such as pDPN.

The robustness of these results was demonstrated by additional analyses that adjusted for demographic and socioeconomic factors, since age, education level, and income may be potential confounding factors that contribute to pain perceptions or HCP interactions. These additional analyses suggest that regardless of socioeconomic status, ethnicity is a general factor in how symptoms associated with pDPN are manifested or perceived.

Additionally, and of potential greater clinical relevance, was the large proportion of African-American and Hispanic populations who were <40 years of age. While it is well-recognized that diabetes disproportionally affects African-Americans and Hispanics [18], to our knowledge this is the first study to suggest that these populations may also have a high prevalence of pDPN symptoms in such a young age group, but a more rigorous epidemiologic study would be needed to corroborate these observations. The overall similarity across ethnicities for time since a diabetes diagnosis further suggests that duration of diabetes is unlikely to meaningfully impact the observed results and their clinical implications.

The differences in symptoms and severity were paralleled by the impact of pain on daily activities on the WPAI:SHP reported by the three populations; the least impairment was consistently reported by Hispanics, and this was significant for Activity impairment vs both other populations, and for Presenteeism vs Caucasians. It should again be noted that the WPAI:SHP responses on work productivity were obtained only from employed respondents, while the activity impairment question was answered by all respondents and was limited to activities other than employment. These observations on the WPAI:SHP are consistent with a recent review suggesting lower rates of activity limitation among Hispanics with pain relative to other cultural groups despite greater pain sensitivity [15]. Among those employed, presenteeism was three times that of absenteeism in all cultural groups, suggesting that this was the primary driver of work impairment, as has been previously reported among patients with chronic pain conditions [5].

Despite the presence of these symptoms and pain of moderate or severe severity in substantial proportions of African-Americans and Hispanics, fewer of these patients reported receiving a pDPN diagnosis than Caucasians. This lower rate of diagnosis may potentially be due, at least in part, to the observations related to interactions of these populations with their HCPs: Fewer African-American and Hispanic patients reported discussing their pain symptoms with their HCP, and there was consistently less comfort with their HCPs in these groups.

These interactions with HCPs are consistent with the disparities in healthcare resource availability and use that have been reported among minority populations and that contribute to the challenge of diagnosis and management of these patients [19]. In particular, Hispanics have reported language and cultural barriers such as the unavailability of Spanish-speaking healthcare providers or interpreters [15, 20]. While these language and cultural barriers may in part account for the lower comfort level of Hispanics with their HCPs in the current study, it should also be noted that African-Americans reported a similarly hard time communicating with their HCPs as Hispanics did.

### Limitations

As with any survey dependent upon respondents, an important limitation is potential selection bias, since patients who agreed to participate may have characteristics and perceptions different from those who refused. A related limitation is that the patient-level data on diagnosis, pain, and symptoms were based on self-report and, as such, may be subject to misunderstanding or misinterpretation of the questions that may result, at least in part, from cultural differences across the populations.

It should also be noted that this study did not capture other factors that may have contributed to patients’ perceptions of their pain experience, such as mood, negative emotions and thoughts, poor pain control, or construals. These factors, as well as others not collected, could be a potential missing source of information that may have contributed to how subjects reported their painful symptoms or interactions with their HCPs, and warrant further evaluation in future studies.

While use of both internet and phone as survey modalities could be criticized, such a design was necessary to reach the target populations, and the inability to disentangle the administration modality from the results across the populations represents another limitation. Lastly, the survey results reflect an unweighted sample, and thus may not necessarily be reflective or representative of the entire general population in the United States. However, the findings provide directional insights that can be used to optimize patient care.

## Conclusions

Significant differences in patient experiences of pDPN symptoms and pain severity were reported across cultural groups including African-Americans, Hispanics, and Caucasians; African-Americans and Hispanics were less likely to experience the same sensations as Caucasian patients and reported lower pain ratings. Further evaluation is needed to determine what may account for these observed differences. Differential rates of pDPN diagnosis and comfort levels with HCPs were also reported in this multicultural population, with the differences providing support for barriers that contribute to disparities in healthcare among specific populations.

These results suggest a need to broaden pDPN educational initiatives among both patients and clinicians. While patient intiatives should especially target multicultural populations, the goals of clinician initiatives should be to increase attention that symptoms may differ among individuals with different cultural backgrounds and to improve patient-HCP dialogue by encouraging discussion of pDPN symptoms and their impact in multicultural settings.

## Additional file

Additional file 1: Multicultural pDPN Research Patient Survey. (DOCX 260 kb)

## Abbreviations

BPI: Brief Pain Inventory
HCP: Healthcare provider
pDPN: Painful diabetic peripheral neuropathy
WPAI:SHP: Work Productivity and Assessment Questionnaire disease-specific version
