# Supplementary material for: Perspectives on the impact of painful diabetic peripheral neuropathy in a multicultural population
Source: Clin Diabetes Endocrinol. 2017 Dec 28;3:12. doi: 10.1186/s40842-017-0051-2 (PMC5745600; doi:10.1186/s40842-017-0051-2)
Supplement: Additional file 1: — Multicultural pDPN Research Patient Survey. (DOCX 260 kb) [file 40842_2017_51_MOESM1_ESM.docx]

| **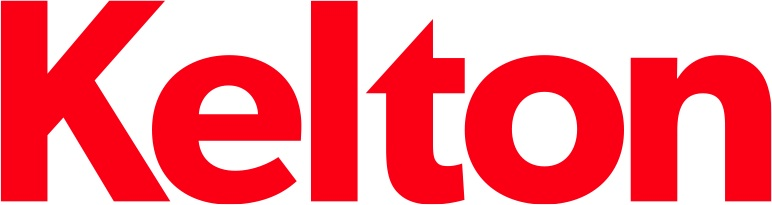** | 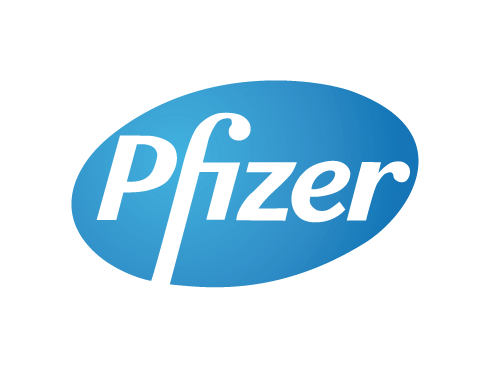**Multicultural pDPN Survey – Patient Audience**  August 2015  Sample: 1,000 American diabetic patients age 18+, diagnosed with pDPN or experiencing at least three symptoms of pDPN  *Oversample to reach total of 500 African Americans and 500 Hispanic Americans (including main sample and oversample)* |
| --- | --- |

**Questionnaire**

**Questionnaire**

**Questionnaire**

This survey is being conducted by Kelton, a leading global insights company. We are not trying to sell you anything, but would like to ask you a few questions for an opinion study. The results may appear in leading media outlets in the coming weeks, but your answers will remain confidential and anonymous.

**QUOTA TABLE:**

| **Total** | **Total** | **1,100** |
| --- | --- | --- |
| Ethnicity |  |  |
| Caucasian | Q5=1 | None – monitor only |
| African American | Q5=2 | None – monitor only |
| Hispanic | Q5=3 | None – monitor only |
| Other | Q5=4-6 | None – monitor only |
| Languages Spoken |  |  |
| English | Q1=1 | None – monitor only |
| Spanish | Q1=2 | None – monitor only |
| Language Taken Survey In |  |  |
| English | Programming variable | None – monitor only |
| Spanish | Programming variable | None – monitor only |
| Region |  |  |
| Northeast | Based on Q73 | None – monitor only |
| South | Based on Q73 | None – monitor only |
| Midwest | Based on Q73 | None – monitor only |
| West | Based on Q73 | None – monitor only |
| Income |  |  |
| Under $75k | Q74=1-4 | None – monitor only |
| $75k+ | Q74=5-7 | None – monitor only |
|  |  |  |
| Gender |  |  |
| Male | Q4=1 | None – monitor only |
| Female | Q4=2 | None – monitor only |
| Age |  |  |
| < 50 | Q3< 50 | None – monitor only |
| 50+ | Q3=50+ | None – monitor only |
| pDPN Diagnosis Status |  |  |
| Diagnosed | Q11=3 | None – monitor only |
| Not | Q11≠3 (0 or missing) | None – monitor only |

**SCREENING QUESTIONS**

1. **(ASK ALL)** What languages do you speak fluently? Please choose all that apply. **(SHOW ENGLISH AND SPANISH TRANSLATION OF THE QUESTION TEXT)** **(RANDOMIZE) (SELECT ALL THAT APPLY)**
2. English
3. Spanish
4. French
5. Mandarin
6. Portuguese
7. Korean
8. Other **(ANCHOR)**

**TERMINATE IF NOT ENGLISH OR SPANISH, TERMINATE IF Q1≠1 OR 2**

1. **(ASK IF SPANISH, Q1=2)** Which language would you prefer to take this survey in? **(SHOW ENGLISH AND SPANISH TRANSLATION OF THE QUESTION TEXT) (RANDOMIZE) (SINGLE RESPONSE)**
2. English
3. Español

**SHOW REMAINDER OF SURVEY IN ENGLISH OR SPANISH IN ACCORDANCE WITH RESPONDENT PREFERENCE**

1. **(ASK ALL)** How old are you? **(RECORD EXACT RESPONSE) TERMINATE IF UNDER 18**
2. **(ASK ALL)** What is your gender? **(SINGLE RESPONSE) (RANDOMIZE)**
3. Male
4. Female
5. **(ASK ALL)** Which of the following best describes your ethnic group? **(SINGLE RESPONSE) (LOCK ORDER)**
   1. White/Caucasian
   2. Black/African-American
   3. Hispanic, or of Latino or Spanish origin
   4. Asian
   5. American Indian or Alaska Native
   6. Other
6. **(ASK ALL)** What is your current employment status? **(SINGLE RESPONSE) (LOCK ORDER)**
7. Employed full time **(CODE AS EMPLOYED)**
8. Employed part time **(CODE AS EMPLOYED)**
9. Self-employed **(CODE AS EMPLOYED)**
10. Full time student
11. Retired
12. Unemployed and looking for work
13. Unemployed and not looking for work at this time
14. Stay at home parent/spouse
15. Disabled
16. **(ASK ALL)** What is your current marital status? **(SINGLE RESPONSE) (LOCK ORDER)**
17. Married or living as married **(CODE AS PARTNERED)**
18. Living with a domestic partner **(CODE AS PARTNERED)**
19. Single, never married
20. Widowed
21. Separated
22. Divorced
23. **(ASK IF SINGLE, WIDOWED, SEPARATED, OR DIVORCED, Q7=3-6)** Are you currently in a committed relationship? **(SINGLE RESPONSE)**
24. Yes **(CODE AS PARTNERED)**
25. No
26. **(ASK ALL)** Do you have any children under the age of 18 who currently live in your home full-time? **(SINGLE RESPONSE)**
    1. Yes **(CODE AS PARENT**)
    2. No, none
27. **(ASK ALL)** Please indicate your level of understanding of each of the following medical conditions. **(RANDOMIZE ROWS ONLY) (SINGLE RESPONSE PER ROW)**

**COLUMNS**

1. I’ve never heard of this condition
2. I’ve heard of this condition, but do not know much about it
3. I know a lot about this condition

**ROWS**

1. Arthritis
2. Diabetes
3. Diabetic Nerve Pain/Painful Diabetic Peripheral Neuropathy (pDPN)
4. Fibromyalgia
5. High Blood Pressure
6. High Cholesterol
7. Insomnia
8. Neuropathy (Non-Diabetic)

**TERMINATE IF HAVE NEVER HEARD OF DIABETES, TERMINATE IF Q10r2=1**

Throughout the survey, we will be referring to healthcare providers – please consider this to include your primary care physician, nurses and any other specialists.

1. **(ASK ALL)** Which of the following conditions, if any, have you ever been diagnosed with by a healthcare provider? Please select all that apply. **(RANDOMIZE) (SELECT ALL THAT APPLY)**

**SHOW CONDITIONS AWARE OF IN PREVIOUS QUESTION (COLUMNS 2 or 3 in Q10)**

1. Arthritis
2. Diabetes
3. Diabetic Nerve Pain/Painful Diabetic Peripheral Neuropathy (pDPN)
4. Fibromyalgia
5. High Blood Pressure
6. High Cholesterol
7. Insomnia
8. Neuropathy (Non-Diabetic)
9. None of the above **(ANCHOR, EXCLUSIVE)**

**TERMINATE IF HAVE NOT BEEN DIAGNOSED WITH DIABETES, IF Q11≠2**

1. **(ASK ALL)** Do you have pain? **(SINGLE RESPONSE) (LOCK ORDER)**
2. Yes
3. No
4. **(ASK ALL)** Have you experienced any kind of pain on a regular basis for at least the past 3 months (or longer)? **(SINGLE RESPONSE) (LOCK ORDER)**
5. Yes
6. No

**IF NOT DIAGNOSED WITH DPN, TERMINATE IF NO PAIN FOR 3+ MONTHS (TERMINATE IF Q11≠3 AND Q13=2)**

1. **(ASK ALL)** Do you have pain other than headaches? **(SINGLE RESPONSE) (LOCK ORDER)**
2. Yes
3. No

**IF NOT DIAGNOSED WITH DPN, TERMINATE IF NO PAIN OTHER THAN A HEADACHE (TERMINATE IF Q11≠3 AND Q14=2)**

1. **(ASK IF Q14=1)** You mentioned you experience pain other than headaches. In particular, in what areas do you experience pain? Please select all that apply. **(RANDOMIZE) (SELECT ALL THAT APPLY)**
2. Feet
3. Toes
4. Hands
5. Fingers
6. Knees
7. Back
8. Arms
9. Legs
10. Somewhere else **(ANCHOR)**

**IF NOT DIAGNOSED WITH DPN, TERMINATE IF NO PAIN IN FEET OR TOES (TERMINATE IF Q11≠3 AND Q15≠1 OR 2)**

1. **(ASK IF PAIN IN MORE THAN ONE AREA, IF MORE THAN ONE PUNCH IS CHOSEN FOR Q15)** You mentioned you experience pain in more than one area. In particular, what area bothers you the most? **(RANDOMIZE) (SINGLE RESPONSE)**

**SHOW AREAS OF PAIN**

1. Feet
2. Toes
3. Hands
4. Fingers
5. Knees
6. Back
7. Arms
8. Legs
9. Somewhere else **(ANCHOR, EXCLUSIVE)**
10. **(ASK IF PAIN IN FEET OR TOES, Q15=1-2)** You mentioned that you experience pain in your feet, toes, hands and/or fingers. In particular, how have you experienced this pain? Select one per group. **(RANDOMIZE ALL) (SINGLE RESPONSE PER GROUPING)**

**Q17_1 – SHOW IF FEET (SHOW IF Q15=1)**

1. My **left** foot only
2. My **right** foot only
3. Both my left and right feet **(ANCHOR)**

**Q17_2 – SHOW IF TOES (SHOW IF Q15=2)**

1. My **left** toes only
2. My **right** toes only
3. Both my left and right toes **(ANCHOR)**

**IF NOT DIAGNOSED WITH DPN, TERMINATE IF PAIN IS NOT IN BOTH FEET OR BOTH TOES (TERMINATE IF Q11≠3 AND Q17_1≠3 AND Q17_2≠3)**

1. **(ASK IF PAIN IN FEET, TOES, HANDS OR FINGERS, Q15=1-4)** You mentioned that you experience pain in your feet/toes or hands/fingers. Which of the following, if any, describe your pain **in those areas over the past week**? **(RANDOMIZE) (SELECT ALL THAT APPLY)**
2. Feels like pins and needles **(programmer note: value=+1 if chosen)**
3. Feels hot/burning **(programmer note: value=+1 if chosen)**
4. Feels numb **(programmer note: value=+1 if chosen)**
5. Feels like electrical shocks **(programmer note: value=+1 if chosen)**
6. It is made worse with the touch of clothing or bed sheets **(programmer note: value=+1 if chosen)**
7. It is limited to my joints **(programmer note: value=-1 if chosen)**
8. None of these **(programmer note: value=0 if chosen)** **(ANCHOR, EXCLUSIVE)**

**IDPainSum. PROGRAMMER: CREATE A SUM OF THE RESPONSE OPTIONS IN Q18**

**TERMINATE IF NOT DIAGNOSED WITH NERVE PAIN/DIABETIC PERIPHERAL NEUROPATHY AND NOT 3+ IN IDPainSum (TERMINATE IF Q11≠3 AND IDPainSum<3)**

**TESTING NOTE: IF DIAGNOSED WITH NERVE PAIN/DIABETIC NEUROPATHY/DIABETIC PERIPHERAL NEUROPATHY, ALWAYS QUALIFIES FOR SURVEY (QUALIFIES IF Q11=3)**

**IF NOT DIAGNOSED WITH** **NERVE PAIN/DIABETIC PERIPHERAL NEUROPATHY, RESPONDENT QUALIFIES IF EXPERIENCE CHRONIC PAIN, EXPERIENCE PAIN OTHER THAN HEADACHE, EXPERIENCE PAIN IN BOTH FEET/TOES AND RESPONDENT’S IDPainSum IS 3+**

**GENERAL NERVE PAIN PERCEPTIONS**

**(SHOW ALL)** Congratulations, you qualify for this survey.

We’re going to start off by asking you about nerve pain, which is also often referred to as painful neuropathy.

**(SHOW IF NOT DIAGNOSED, SHOW IF Q11≠3)** For the purposes of this survey, please consider “nerve pain” to be pain *other than a headache* that feels like a burning sensation, electrical shocks, tingling/prickling, shooting pain, radiating pain, stinging, throbbing or stabbing pain.

1. **(ASK ALL)** In general, how would you describe your knowledge of nerve pain? **(SINGLE RESPONSE) (LOCK ORDER) (DISPLAY AS SLIDER)**
2. No knowledge
3. Modest level of knowledge
4. Moderate level knowledge
5. High level of knowledge
6. **(ASK ALL)** What would you consider to be the cause(s) of nerve pain? **(SELECT ALL) (RANDOMIZE)**
   1. Damaged nerves
   2. Genetics
   3. Another condition or disease (e.g. cancer, diabetes, HIV)
   4. Aging in general
   5. Poor diet
   6. Not exercising enough
   7. Other **(ANCHOR)**
   8. I’m not sure **(ANCHOR, EXCLUSIVE)**
7. **(ASK ALL)** Please tell us how strongly you agree or disagree with each of the following statements about nerve pain and diabetes. **(SINGLE RESPONSE PER ROW) (RANDOMIZE ROWS)**

**COLUMNS**

- 1. Strongly disagree
  2. Somewhat disagree
  3. Somewhat agree
  4. Strongly agree

**ROWS**

1. Nerve pain is a common complication of diabetes
2. Nerve pain is an expected part of what people with diabetes have to deal with
3. Nerve pain is unavoidable for those who have diabetes
4. There is no cure for nerve pain
5. Having chronically high blood sugar can damage your nerves

**SYMPTOMS, SEVERITY & IMPACT**

Shifting gears, we’d like to ask you about the symptoms you experience and the impact of the pain in your feet and/or hands on your ability to work and perform regular activities. Earlier, you mentioned you experience pain in your feet and/or hands. This may be a type of nerve pain. **Throughout the survey, when we mention “nerve pain” we are referring to pain in your feet and/or hands.**

1. **(ASK ALL)** Please indicate your experience with each of the following symptoms in your feet and/or hands. **(RANDOMIZE ALL) (SINGLE RESPONSE PER ROW)**

**COLUMNS**

1. Have never experienced
2. Have experienced, but do not currently experience on most days
3. Currently experience on most days

**ROWS**

1. Numbness
2. Pins and needles
3. Pain or discomfort at night
4. Tingling or prickling sensation
5. Sensitivity to touch
6. Burning pain or sensation
7. Shooting pain
8. Stinging
9. Stabbing pain
10. Electric shock-like symptoms or sudden pain attacks

**PROGRAMMING NOTE: IF RESPONDENT NOTED A SYMPTOM IN Q18, DISPLAY AN ERROR MESSAGE IF THEY DO NOT INDICATE EVER EXPERIENCING THAT SYMPTOM IN Q22**

1. **(ASK ALL)** On average, how often do you experience nerve pain in your feet and/or hands? **(SINGLE RESPONSE) (LOCK ORDER)**
   1. Rarely
   2. Sometimes
   3. Most of the time
   4. Always
2. **(ASK ALL)** About how long have you been experiencing nerve pain in your feet and/or hands? **(NUMERIC OPEN END)**

**_____** year(s) **(PROGRAMMER: MINIMUM 1; DATA PROCESSOR: MAXIMUM NOT TO EXCEED RESPONDENT’S AGE IN Q3)**

Less than 1 year

1. **(ASK ALL)** Throughout our lives, most of us have had pain from time to time (such as minor headaches, sprains and toothaches). Have you had pain other than these kinds of pain today? **(SINGLE RESPONSE) (LOCK ORDER)**
   1. Yes
   2. No
2. **(ASK ALL)** Please rate your pain by selecting the one number that best describes your pain at its worst in the last 24 hours. **(SINGLE RESPONSE) (LOCK ORDER) (DISPLAY SCALE AS A HORIZONTAL SLIDER)**
   1. 0 – No Pain
   2. 1
   3. 2
   4. 3
   5. 4
   6. 5
   7. 6
   8. 7
   9. 8
   10. 9
   11. 10 – Pain as bad as you can imagine

1. **(ASK ALL)** Please rate your pain by selecting the one number that best describes your pain at its least in the last 24 hours. **(SINGLE RESPONSE) (LOCK ORDER) (DISPLAY SCALE AS A HORIZONTAL SLIDER)**
   1. 0 – No Pain
   2. 1
   3. 2
   4. 3
   5. 4
   6. 5
   7. 6
   8. 7
   9. 8
   10. 9
   11. 10 – Pain as bad as you can imagine

1. **(ASK ALL)** Please rate your pain by selecting the one number that best describes your pain on the average. **(SINGLE RESPONSE) (LOCK ORDER) (DISPLAY HORIZONTALLY)**
   1. 0 – No Pain
   2. 1
   3. 2
   4. 3
   5. 4
   6. 5
   7. 6
   8. 7
   9. 8
   10. 9
   11. 10 – Pain as bad as you can imagine

1. **(ASK ALL)** Please rate your pain by selecting the one number that tells how much pain you have right now. **(SINGLE RESPONSE) (LOCK ORDER) (DISPLAY HORIZONTALLY)**
   1. 0 – No Pain
   2. 1
   3. 2
   4. 3
   5. 4
   6. 5
   7. 6
   8. 7
   9. 8
   10. 9
   11. 10 – Pain as bad as you can imagine

1. **(ASK ALL)** In the last 24 hours, how much relief have pain treatments or medications provided? Please select the one percentage that most shows how much relief you have received. **(SINGLE RESPONSE) (LOCK ORDER) (DISPLAY HORIZONTALLY)**
   1. 0% - No relief
   2. 10%
   3. 20%
   4. 30%
   5. 40%
   6. 50%
   7. 60%
   8. 70%
   9. 80%
   10. 90%
   11. 100% - Complete relief
2. **(ASK ALL)** Select the one number that describes how, during the past 24 hours, pain has interfered with your: **(SINGLE RESPONSE PER ROW) (LOCK ORDER OF COLUMNS; RANDOMIZE ROWS)**

**COLUMNS**

- 1. 0 – Does not interfere
  2. 1
  3. 2
  4. 3
  5. 4
  6. 5
  7. 6
  8. 7
  9. 8
  10. 9
  11. 10 – Completely interferes

**ROWS**

1. General activity
2. Mood
3. Walking ability
4. Normal work (includes both work outside the home and housework)
5. Relations with other people
6. Sleep
7. Enjoyment of life

Now, please think about the past year.

1. **(ASK ALL)** Please rate the severity of your nerve pain by selecting the one number that best describes the pain in your feet and/or hands on average within the past year. **(SINGLE RESPONSE) (DISPLAY SCALE AS A HORIZONTAL SLIDER)**
   1. 0 – No Pain
   2. 1
   3. 2
   4. 3
   5. 4
   6. 5
   7. 6
   8. 7
   9. 8
   10. 9
   11. 10 – Pain as bad as you can imagine

1. **(ASK ALL)** Within the past year how, if at all, nerve pain in your feet and/or hands has interfered with your: **(SINGLE RESPONSE PER ROW) (RANDOMIZE ROWS) (DISPLAY SCALE AS A HORIZONTAL SLIDER)**

**COLUMNS**

- 1. 0 – Does not interfere
  2. 1
  3. 2
  4. 3
  5. 4
  6. 5
  7. 6
  8. 7
  9. 8
  10. 9
  11. 10 – Completely interferes

**ROWS**

1. General activity
2. Mood
3. Walking ability
4. Relations with other people
5. Sleep
6. Enjoyment of life
7. Exercise
8. Housework
9. Cooking
10. Hobbies
11. Ability to travel
12. Ability to participate in leisurely physical activities (e.g. dancing, playing sports)
13. **(SHOW IF EMPLOYED, IF Q6=1-3)** Work
14. **(ASK ALL)** Please tell us how strongly you agree or disagree with each of the following statements about the nerve pain in your feet and/or hands.  **(SINGLE RESPONSE PER ROW) (RANDOMIZE ROWS ONLY) (DISPLAY SCALE AS A HORIZONTAL SLIDER)**

**COLUMNS**

- 1. Strongly disagree
  2. Somewhat disagree
  3. Somewhat agree
  4. Strongly agree

**ROWS**

1. My nerve pain is under control
2. I could better manage my nerve pain if I had access to more information (e.g., treatment options, disease resources) on it
3. Nerve pain impacts my day to day life more than any of my other symptoms
4. I am hesitant to plan activities because I fear that nerve pain will prevent me from participating
5. I feel that I am doing everything I can to reduce my nerve pain (through treatment or other management techniques)
6. My nerve pain makes it difficult for me to live a healthy lifestyle (e.g. exercise, eat healthy)
7. **(IF DPN DIAGNOSED, Q11=3)** My nerve pain makes me feel like I’m not successfully managing my diabetes
8. **(IF DPN DIAGNOSED, Q11=3)** My nerve pain has improved since speaking with my healthcare provider about my pain
9. **(IF DPN DIAGNOSED, Q11=3)** When I was first diagnosed, I expected that I’d be able to reduce my nerve pain more than I actually can

The following activities ask about the effect of your nerve pain on your ability to work and perform regular activities.

**Q34A. (ASK ALL)** Are you currently employed (working for pay)? **(SINGLE RESPONSE)**

1. Yes
2. No

**PROGRAMMER: DISPLAY ERROR MESSAGE IF RESPONDENT PREVIOUSLY INDICATED THEY ARE EMPLOYED BUT RESPOND NO IN Q35. DISPLAY ERROR MESSAGE IF RESPONDENT PREVIOUSLY INDICATED THEY ARE NOT EMPLOYED BUT RESPOND YES IN Q35.**

These next questions are about the past seven days, not including today.

1. **(ASK IF EMPLOYED, Q6=1-3)** During the past seven days, how many hours did you miss from work because of problems associated with your pain? Include hours you missed on sick days, times you went in late, left early, etc., because of your pain. **(NUMERIC OPEN END) (MINIMUM 0) (MAXIMUM 168)**

________ hour(s)

1. **(ASK IF EMPLOYED, Q6=1-3)** During the past seven days, how many hours did you miss from work because of any other reason, such as vacation or holidays? **(NUMERIC OPEN END) (MINIMUM 0) (MAXIMUM 168)**

________ hour(s)

1. **(ASK IF EMPLOYED, Q6=1-3)** During the past seven days, how many hours did you actually work? **(NUMERIC OPEN END) (MINIMUM 0) (MAXIMUM 168)**

________ hour(s)

1. **(ASK IF EMPLOYED, Q6=1-3)** During the past seven days, how much did your pain affect your productivity while you were working? **(DISPLAY SCALE AS HORIZONTAL SLIDER)**

Think about days you were limited in the amount or kind of work you could do, days you accomplished less than you would like, or days you could not do your work as carefully as usual. If pain affected your work only a little, choose a low number. Choose a high number if pain affected your work a great deal. Consider only how much pain affected productivity while you were working.

- 1. 0 – Pain had no effect on my work
  2. 1
  3. 2
  4. 3
  5. 4
  6. 5
  7. 6
  8. 7
  9. 8
  10. 9
  11. 10 – Pain completely prevented me from working

1. **(ASK ALL)** During the past seven days, how much did your pain affect your ability to do your regular daily activities, other than work at a job? **(DISPLAY SCALE AS HORIZONTAL SLIDER)**

By regular activities, we mean the usual activities you do, such as work around the house, shopping, childcare, exercising, studying, etc. Think about the times you were limited in the amount or kind of activities you could do and times you accomplished less than you would like. If pain affected your activities only a little, choose a low number. Choose a high number if pain affected your activities a great deal. Consider only how much pain affected your ability to do your regular daily activities, other than work at a job.

- 1. 0 – Pain had no effect on my daily activities
  2. 1
  3. 2
  4. 3
  5. 4
  6. 5
  7. 6
  8. 7
  9. 8
  10. 9
  11. 10 – Pain completely prevented me from doing my daily activities

**SYMPTOM MANAGEMENT**

For this next set of questions, we’re interested in learning about the ways in which you manage and treat your nerve pain. As a reminder, when we mention “nerve pain” we are referring to the pain in your feet and/or hands.

1. **(ASK ALL)** Which of the following statements about nerve pain treatments, if any, do you agree with? **(SELECT ALL) (RANDOMIZE)**

Treatment can…

1. Provide long-term relief from my nerve pain
2. Provide short-term relief from my nerve pain
3. Repair or reverse the cause(s) of my nerve pain
4. Not help this condition **(ANCHOR, EXCLUSIVE)**
5. None of these **(ANCHOR, EXCLUSIVE)**
6. **(ASK ALL)** Please indicate your experience with each of the following treatments as they relate to the nerve pain in your feet and/or hands. **(SINGLE RESPONSE PER ROW) (RANDOMIZE ROWS ONLY)**

**COLUMNS**

1. Never used
2. Used previously, but do not currently use
3. Currently use

**ROWS**

- - - 1. **(SHOW IF DIAGNOSED, Q11=3)** Prescription medication
      2. Over-the-counter medication
      3. Home remedies (e.g. herbal teas, ice packs)
      4. Acupuncture
      5. Physical therapy
      6. Massage therapy
      7. Exercise
      8. Healthy diet
      9. Electrical stimulation
      10. Topical treatments (e.g. creams, lotions, gels, patches)
      11. Other treatment **(ANCHOR)**

1. **(ASK IF TRIED PRESCRIPTION RX,Q41r1=2-3)** You mentioned you have tried prescription medication to treat your nerve pain. Approximately how much time passed in between your initial nerve pain diagnosis and your healthcare provider prescribing medication for you? **(SINGLE RESPONSE) (LOCK ORDER)**
   1. Less than 1 month
   2. 1 month to less than 6 months
   3. 6 months to less than 1 year
   4. 1 year to 4 years
   5. 5 years to 10 years
   6. Over 10 years
2. **(ASK ALL)** Please tell us how strongly you agree or disagree with each of the following statements about your current treatment plan for the nerve pain in your feet and/or hands. **(SINGLE RESPONSE PER ROW) (RANDOMIZE ROWS ONLY)**

**COLUMNS**

- 1. Strongly disagree
  2. Somewhat disagree
  3. Somewhat agree
  4. Strongly agree

**ROWS**

1. I feel as if my doctor and I don’t agree on my treatment options
2. Through treatment, I think there could be a point where my pain could be significantly reduced
3. I trust my doctor to make the best treatment decisions for me

**SUPPORT NETWORK**

Next, we’d like to ask a few questions about the role your support network (e.g. family, friends) plays in your life.

1. **(ASK ALL)** In general, how involved are your family and/or friends in your health? **(SINGLE RESPONSE) (LOCK ORDER) (SHOW AS SLIDER)**
   1. 1- Not at all involved
   2. 2
   3. 3
   4. 4
   5. 5- Very involved
2. **(ASK ALL)** Who have you ever talked to about the nerve pain in your feet and/or hands? **(SELECT ALL) (RANDOMIZE)**
   - 1. Family members, such as parents, siblings and other close relatives
     2. Friends
     3. Healthcare provider(s)
     4. **(SHOW IF EMPLOYED,Q6=1-3)** Co-workers
     5. **(SHOW IF EMPLOYED & NOT SELF-EMPLOYED,Q6=1-2)** Boss
     6. **(SHOW IF PARTNERED,Q7=1-2 OR Q8=1)** Significant other
     7. **(SHOW IF PARENT,Q9=1)** My children
     8. Other **(ANCHOR)**
     9. No one **(ANCHOR, EXCLUSIVE)**
3. **(ASK IF FAMILY OR FRIENDS INVOLVED, Q44=2-5)** You mentioned that your family/friends are involved in your health. Specifically, what have they done to help you manage your health? **(SELECT ALL) (RANDOMIZE)**
   1. Provided recommendations or advice
   2. Came to a doctor visit with me
   3. Helped me manage my medications
   4. Helped me take care of household responsibilities (e.g. cooking, cleaning)
   5. Researched/provided me with information on my condition(s)
   6. Other **(ANCHOR)**

**HCP RELATIONSHIP**

Switching topics, we’d like to ask you some questions about the types of healthcare providers you see and your experiences with these healthcare providers.

1. **(ASK ALL)** Please indicate about how often, if at all, you visit each of the following types of healthcare providers. Please select the option that comes closest. **(RANDOMIZE ROWS ONLY) (SINGLE RESPONSE PER ROW)**

**COLUMNS**

- 1. Once a week
  2. Once a month
  3. Once every three months
  4. Once every six months
  5. Once a year
  6. Less often than once a year
  7. Never visit

**ROWS**

- - - 1. Primary Care Physician
      2. Nurse, Nurse Practitioner or Physician Assistant
      3. Physical Therapist
      4. Acupuncturist
      5. Massage Therapist
      6. Neurologist
      7. Pain Management Specialist
      8. Endocrinologist
      9. **(SHOW IF FEMALE, Q4=2)** Gynecologist
      10. Other healthcare provider **(ANCHOR)**

As a reminder, when we mention “nerve pain” we are referring to the pain in your feet and/or hands**.**

1. **(ASK ALL)** Which healthcare providers do you see for the treatment or management of…? **(SELECT ALL PER COLUMN) (RANDOMIZE ALL)**

**COLUMNS**

1. Your diabetes
2. The nerve pain in your feet and/or hands

**ROWS – SHOW HCPs EVER VISITED**

- - 1. Primary Care Physician
    2. Nurse, Nurse Practitioner or Physician Assistant
    3. Physical Therapist
    4. Acupuncturist
    5. Massage Therapist
    6. Neurologist
    7. Pain Management Specialist
    8. Endocrinologist
    9. Gynecologist
    10. Other healthcare provider **(ANCHOR)**
    11. None **(ANCHOR, EXCLUSIVE PER COLUMN)**

1. **(ASK IF VISIT HCP FOR NERVE PAIN OR DIABETES, Q48c1=1-10 or Q48c2=r1-10)** And which do you consider to be your primary healthcare provider in the treatment of…? **(RANDOMIZE) (SINGLE RESPONSE PER COLUMN)**

**COLUMNS**

1. **Your diabetes** **(SHOW IF Q48c1=r1-10)**
2. **The nerve pain in your feet and/or hands** **(SHOW IF Q48c2=r1-10)**

**ROWS – SHOW HCPs EVER VISITED IN Q48**

- - 1. Primary Care Physician
    2. Nurse, Nurse Practitioner or Physician Assistant
    3. Physical Therapist
    4. Acupuncturist
    5. Massage Therapist
    6. Neurologist
    7. Pain Management Specialist
    8. Endocrinologist
    9. Gynecologist
    10. Other healthcare provider **(ANCHOR)**

**PrimaryHCP. AUTOCODE: SELECT IF RESPONDENT CONSIDERS THIS TYPE OF PROVIDER AS PRIMARY FOR THEIR NERVE PAIN IN Q49c2**

1. Primary Care Physician
2. Nurse, Nurse Practitioner or Physician Assistant
3. Physical Therapist
4. Acupuncturist
5. Massage Therapist
6. Neurologist
7. Pain Management Specialist
8. Endocrinologist
9. Gynecologist
10. Other
11. None **(NO PRIMARY HCP IN Q49c2)**

***PROGRAMMING NOTE: FOR THE FOLLOWING PIPING, PIPE IN PROVIDER TYPE IF SELECTED IN THE PRIMARY HCP AUTOCODE. IF ‘OTHER’ IN PRIMARY HCP AUTOCODE, PIPE IN:*** healthcare provider

**(SHOW IF VISIT ANY OF THE PROVIDED HCP TYPES FOR NERVE PAIN, Q49c2=1-9)** For the remainder of the survey, we’re going to ask you about the **[PIPE IN THE HCP THEY CONSIDER TO BE PRIMARY FOR NERVE PAIN FROM PrimaryHCPAutocode]** you see, whom you mentioned you consider the primary healthcare provider in the management of the nerve pain in your feet and/or hands.

**(SHOW IF PRIMARY HCP FOR NERVE PAIN IS ‘OTHER’ IN AUTOCODE, PrimaryHCP=10)** For the remainder of the survey, we’re going to ask you about the healthcare provider you consider to be primary in the management of the nerve pain in your feet and/or hands.

**(SHOW IF DO NOT VISIT ANY HCP FOR NERVE PAIN BUT VISIT ANY OF THE PROVIDED HCP TYPES FOR DIABETES, PrimaryHCP=11 AND Q48c1=1-9)** For the remainder of the survey, we’re going to ask you about the **[PIPE IN THE HCP THEY CONSIDER TO BE PRIMARY FOR DIABETES, RESPONSE IN Q48c1]** you see, whom you mentioned you consider the primary healthcare provider in the management of your diabetes.

**(SHOW DO NOT VISIT ANY HCP FOR PAIN BUT PRIMARY HCP FOR DIABETTES IS ‘OTHER’, PrimaryHCP=11 AND Q48c1=10)** For the remainder of the survey, we’re going to ask you about the healthcare provider you consider to be primary in the management of your diabetes.

1. **(ASK IF VISIT HCP FOR PAIN OR DIABETES, Q48c1=1-10 or Q48C2=r1-10)** Where do you most often visit your primary healthcare provider? **(SINGLE RESPONSE) (RANDOMIZE)**
   1. At a doctor’s office
   2. At a clinic
   3. In an emergency room
   4. Other **(ANCHOR)**

As a reminder, when we mention “nerve pain” we are referring to the pain in your feet and/or hands**.**

1. **(ASK IF VISIT HCP FOR PAIN OR DIABETES, Q48c1=1-10 or Q48C2=r1-10)** Which of the following topics, if any, have you ever discussed with your primary healthcare provider? **(SELECT ALL) (RANDOMIZE)**
   1. The nerve pain you experience in your feet and/or hands
   2. Symptoms experienced (other than pain)
   3. Medications you’re currently taking
   4. Importance of exercise/staying active as a diabetes management approach
   5. Importance of nutrition/diet as a diabetes management approach
   6. Your vital signs (e.g. blood pressure)
   7. Your bloodwork results (e.g. blood sugar, cholesterol levels)
   8. Other pain you experience (not in your feet and/or hands) (ANCHOR)
   9. None of these **(ANCHOR, EXCLUSIVE)**
2. **(ASK IF EVER DISCUSSED NERVE PAIN W/ HCP, Q51=1)** You mentioned you’ve discussed the nerve pain in your feet and/or hands with your primary healthcare provider. Specifically, what did you talk about? **(SELECT ALL) (RANDOMIZE)**
   1. The severity of your pain
   2. The symptoms of pain you experience (e.g. tingling, stabbing)
   3. Pain management expectations, such as how to set and track goals
   4. Medication-related treatment options for pain (over-the-counter or prescription)
   5. Alternative therapy options for pain, such as massage therapy or acupuncture
   6. Impact of your pain on your relationships with other people
   7. Impact of your pain on your ability to carry out basic functions (e.g. general activity, housework)
   8. Other **(ANCHOR)**
3. **(ASK IF DISCUSSED TREATMENT, Q52=4-5)** You mentioned you have discussed nerve pain treatment options with your healthcare provider. In these discussions, who initiated the conversation? **(SINGLE RESPONSE) (RANDOMIZE)**
4. Myself
5. My primary healthcare provider
6. Can’t remember **(ANCHOR)**
7. **(ASK IF DISCUSSED NERVE PAIN W/ HCP, Q51=1)** You mentioned you have discussed the nerve pain in your hands and/or feet with your primary healthcare provider. Who initiated the conversation? **(SINGLE RESPONSE) (RANDOMIZE)**
8. Myself
9. My primary healthcare provider
10. Can’t remember **(ANCHOR)**
11. **(ASK IF DISCUSSED NERVE PAIN W/ HCP, Q51=1)** And how long ago did you **first** speak with your healthcare provider about the nerve pain in your hands and/or feet? **(NUMERIC OPEN END)**

**______** year(s) ago **(PROGRAMMER: MINIMUM 1; DATA PROCESSOR: MAXIMUM NOT TO EXCEED LENGTH OF RESPONDENT’S PAIN IN Q24)**

Less than 1 year ago

1. **(ASK IF DPN DIAGNOSED, Q11=3)** When you initially spoke with your healthcare provider about your nerve pain, when did this conversation happen? **(SINGLE RESPONSE) (RANDOMIZE)**
2. At the time of my official diagnosis
3. Before my official diagnosis
4. **(ASK IF BEFORE DIAGNOSIS, Q56=2)** You mentioned the initial conversation you had with your healthcare provider about your nerve pain happened before your official diagnosis. Approximately how much time passed in between the initial conversation and your diabetic nerve pain diagnosis? **(SINGLE RESPONSE) (LOCK ORDER)**
   1. Less than 1 month
   2. 1 month to less than 6 months
   3. 6 months to less than 1 year
   4. 1 year to 4 years
   5. 5 years to 10 years
   6. Over 10 years
5. **(ASK ALL) [IF HAVE NEVER TALKED ABOUT PAIN Q51≠1, SHOW:** What, if anything, might encourage you to talk with your healthcare provider about the nerve pain in your hands and/or feet?**] [IF HAVE EVER TALKED ABOUT PAIN Q51=1, SHOW:** What, if anything, initially encouraged you to talk with your healthcare provider about the nerve pain in your hands and/or feet?**] (SELECT ALL) (RANDOMIZE)**
   1. **(IF Q51≠1, SHOW)** If my pain were to interfere with my ability to carry out every day activities/ **(IF Q51=1, SHOW)** My pain was interfering with my ability to carry out every day activities
   2. **(IF Q51≠1, SHOW)**If my healthcare provider asked me about my pain/**(IF Q51=1, SHOW)** My healthcare provider asked me about my pain
   3. **(IF Q51≠1, SHOW)**If it were easier to communicate with my healthcare provider about my pain/**(IF Q51=1, SHOW)** It was easy to communicate with my healthcare provider about my pain
   4. **(IF Q51≠1, SHOW)**If I had more information about this type of pain/**(IF Q51=1, SHOW)** I was provided with information about this type of pain
   5. **(IF Q51≠1, SHOW)**If I were to have a friend or family member with me during a healthcare provider visit/**(IF Q51=1, SHOW)** I had a friend or family member come with me to a healthcare provider visit
   6. **(IF Q51≠1, SHOW)**If my pain were to impact my ability to work/**(IF Q51=1, SHOW)** My pain impacted my ability to work
   7. **(IF Q51≠1, SHOW)**If my pain were to impact my ability to sleep/**(IF Q51=1, SHOW)** My pain impacted my ability to sleep
   8. **(IF Q51≠1, SHOW)**If I knew my healthcare provider would be able to recommend a treatment/**(IF Q51=1, SHOW)** I knew my healthcare provider would be able to recommend a treatment
   9. Other **(ANCHOR)**
   10. Nothing **(ANCHOR, EXCLUSIVE)**
6. **(ASK ALL) [IF HAVE NEVER TALKED ABOUT PAIN SHOW, Q51≠1:**  To what degree does each of the following reasons **discourage you** from talking to your healthcare provider about the nerve pain in your hands and/or feet?**]**  **[IF HAVE EVER TALKED ABOUT PAIN SHOW, Q51=1:** To what degree did each of the following reasons **discourage you** from talking to your healthcare provider about the nerve pain in your hands and/or feet when you first began experiencing symptoms?**]** **(SINGLE RESPONSE PER ROW) (RANDOMIZE ROWS ONLY)**

**COLUMNS**

1. Not at all
2. A little bit
3. A lot

**ROWS**

- - - 1. **(IF Q51≠1, SHOW)** The pain doesn’t seem severe enough/**(IF Q51=1, SHOW)** The pain didn’t seem severe enough
      2. **(IF Q51≠1, SHOW)** Concerned about potentially needing surgery/**(IF Q51=1, SHOW)** Was concerned about potentially needing surgery
      3. **(IF Q51≠1, SHOW)** Concerned about potentially needing medication/**(IF Q51=1, SHOW)** Was concerned about potentially needing medication
      4. **(IF Q51≠1, SHOW)** I feel as if my healthcare provider wouldn’t be able to help/**(IF Q51=1, SHOW)** I felt as if my healthcare provider wouldn’t be able to help
      5. **(IF Q51≠1, SHOW)** Concerned that a healthcare provider might tell me something I don’t want to hear/**(IF Q51=1, SHOW)** Was concerned that a healthcare provider might tell me something I don’t want to hear
      6. **(IF Q51≠1, SHOW)** Concerned about the financial expenses it could cause/**(IF Q51=1, SHOW)** Was concerned about the financial expenses it could cause
      7. **(IF Q51≠1, SHOW)** I can handle the pain on my own/**(IF Q51=1, SHOW)** I felt as if I could handle the pain on my own
      8. **(IF Q51≠1, SHOW)** I find it difficult to communicate with my healthcare provider about my symptoms/**(IF Q51=1, SHOW)** I found it difficult to communicate with my healthcare provider about my symptoms
      9. **(IF Q51≠1, SHOW)** I’m not comfortable enough with my healthcare provider to discuss my pain/**(IF Q51=1, SHOW)** I wasn’t comfortable enough with my healthcare provider to discuss my pain
      10. **(IF Q51≠1, SHOW)** I don’t want to complain/**(IF Q51=1, SHOW)** I didn’t want to complain
      11. **(IF Q51≠1, SHOW)** I don’t think there’s anything that could help me/**(IF Q51=1, SHOW)** I didn’t think there was anything that could help me
      12. **(IF Q51≠1, SHOW)** The pain does not interfere with my daily living/**(IF Q51=1, SHOW)** The pain did not interfere with my daily living
      13. **(SHOW ALL)** I prioritize other topics when speaking with my healthcare provider

1. **(ASK ALL) [IF HAVE NEVER TALKED ABOUT PAIN SHOW, Q51≠1:** How severe would the nerve pain in your hands and/or feet have to be for you to speak with your healthcare provider about it?**] [IF HAVE EVER TALKED ABOUT PAIN SHOW, Q51=1:** When you initially spoke with your healthcare provider about the nerve pain in your hands and/or feet, what was your level of pain?**]** **(SINGLE RESPONSE) (LOCK ORDER)**
   1. Mild pain
   2. Moderate pain
   3. Severe pain
2. **(ASK ALL)** How strongly do you agree or disagree with each of the following statements? **(SINGLE RESPONSE PER ROW) (RANDOMIZE ROWS ONLY) (DISPLAY AS SLIDER)**

**COLUMNS**

- 1. Strongly disagree
  2. Somewhat disagree
  3. Somewhat agree
  4. Strongly agree

**ROWS**

1. I tend to wait to discuss symptoms I’m experiencing until I am very comfortable with a healthcare provider
2. I find my primary healthcare provider relatable
3. My primary healthcare provider always has my best interest in mind
4. I defer to my doctor to make the best decisions for my health
5. I feel the need to take an active role in managing my own health
6. I have a hard time communicating my symptoms to my healthcare provider
7. My primary healthcare provider understands my culture
8. I feel there is strong trust between my healthcare provider and myself
9. **(SHOW IF DOESN’T SPEAK ENGLISH, Q1≠1)** Language creates a barrier between me and my healthcare provider
10. **(SHOW IF EVER DISCUSSED PAIN W/ HCP, Q51=1)** I wish I had spoken with my primary healthcare provider about my symptoms/nerve pain sooner

**CUSTOM DEMOGRAPHIC QUESTIONS**

Thank you for your time. The following questions are for statistical classification only.

1. **(ASK ALL)** How many prescription medications are you currently taking in total? **(NUMERIC OPEN END)**

__ medication(s) **(RANGE: 1-25)**

No medications

1. **(ASK ALL)** How many years ago were you first diagnosed with diabetes by a healthcare professional? **(NUMERIC OPEN END)**

__ year(s) ago **(PROGRAMMER: MINIMUM 1; DATA PROCESSOR: MAXIMUM NOT TO EXCEED RESPONDENT’S AGE IN Q3)**

Less than 1 year ago

1. **(ASK IF DIAGNOSED, Q11=3)** How many years ago were you first diagnosed with diabetic nerve pain by a healthcare professional? **(NUMERIC OPEN END)**

__ year(s) ago **(PROGRAMMER: MINIMUM 1; DATA PROCESSOR: MAXIMUM NOT TO EXCEED LENGTH OF RESPONDENT’S PAIN, Q24)**

Less than 1 year ago

1. **(ASK ALL)** Which of the following best describes the type of medical insurance you currently have? **(SINGLE RESPONSE) (RANDOMIZE)**
   1. Medicare
   2. Medicaid
   3. Private insurance (individual or family)
   4. Other **(ANCHOR)**
   5. Do not currently have medical insurance **(ANCHOR)**
2. **(ASK IF LANGUAGE OTHER THAN ENGLISH, Q1=2-7)** Are the language(s) spoken at your primary doctor’s office…? **(SINGLE RESPONSE) (RANDOMIZE)**
   1. The same language(s) you speak
   2. Different from the language(s) you speak

1. **(ASK ALL)** Which of the following statements better describes you? **(SINGLE RESPONSE) (RANDOMIZE)**
   1. I have a consistent primary healthcare provider I usually see when I visit the doctor
   2. I usually see a different primary healthcare provider when I visit the doctor

1. **(ASK ALL)** How frequently do you receive foot exams from your healthcare provider? **(SINGLE RESPONSE) (LOCK ORDER)**
   1. Every visit
   2. Every few visits
   3. Less often than every few visits
   4. Never
2. **(ASK IF UNEMPLOYED/NOT LOOKING FOR WORK OR DISABLED, Q6=7 OR 9)** Earlier, you mentioned you are not currently working. Which of the following best describes why? **(SINGLE RESPONSE) (LOCK ORDER)**
   1. The pain in my feet and/or hands prevents me from working
   2. Another condition prevents me from working
   3. I choose not to work
   4. Other

**STANDARD DEMOGRAPHIC QUESTIONS**

1. **(ASK ALL)** What is your highest level of formal education? **(SINGLE RESPONSE) (LOCK ORDER)**
2. High school – no degree
3. High school – degree
4. Some college – no degree
5. Graduated college – Associate’s degree (2 years)
6. Graduated college – Bachelor’s degree (4 years)
7. Post-graduate degree – MA, MBA, MD, PhD, etc.
8. **(ASK ALL)** Do you live in the city, the suburbs, or in a rural area? **(SINGLE RESPONSE) (RANDOMIZE)**
9. Small city/town (less than 50,000 population)
10. City/urban area (greater than or equal to 50,000 population)
11. Suburbs
12. Rural area (for example: in a very small town in the country or on a farm)
13. **(ASK ALL)** What state do you live in? **(DROP DOWN MENU, 50 STATES PLUS DC)**
14. **(ASK ALL)** Specifically, what is the zip code where you live? **(NUMERIC OPEN END) (ALLOW ONLY 5 DIGIT ZIP CODE)**
15. **(ASK ALL)** Which of the following categories includes your total household income (before taxes) for last year? **(SINGLE RESPONSE) (LOCK ORDER)**
16. Less than $25,000
17. $25,000-$34,999
18. $35,000-$49,999
19. $50,000-$74,999
20. $75,000-$99,999
21. $100,000-$149,999
22. $150,000 or more
23. **(ASK IF HISPANIC OR LATINO AND SPEAK SPANISH, Q5=3 & Q1=2)** When you are at home, speaking with family and friends, would you say you prefer to speak …? **(SINGLE RESPONSE) (LOCK ORDER)**
24. Only in Spanish **(PROGRAMMER: ASSIGN 0 POINTS)**
25. Mostly in Spanish, but some English **(PROGRAMMER: ASSIGN 2 POINTS)**
26. Spanish and English equally **(PROGRAMMER: ASSIGN 4 POINTS)**
27. Mostly English, but some Spanish **(PROGRAMMER: ASSIGN 6 POINTS)**
28. Only in English **(PROGRAMMER: ASSIGN 8 POINTS)**
29. **(ASK IF HISPANIC OR LATINO AND SPEAK SPANISH, Q5=3 & Q1=2)** Please indicate how well you speak English and Spanish. **(SINGLE RESPONSE PER ROW) (RANDOMIZE ROWS) (DATA PROCESSOR: FLAG IF RESPONDENT SELECTS ‘NOT AT ALL’ FOR SPANISH)**

**ROWS**

- 1. I speak English…
  2. I speak Spanish…

**COLUMNS**

1. Not at all
2. Very little
3. A little
4. Well
5. Very well
6. **(ASK IF HISPANIC OR LATINO AND SPEAK SPANISH, Q5=3 & Q1=2)** In what language do you prefer to communicate? **(SINGLE RESPONSE) (RANDOMIZE)**
7. English
8. Spanish
9. No preference **(ANCHOR)**
10. **(ASK IF HISPANIC OR LATINO AND SPEAK SPANISH, Q5=3 & Q1=2)** Were you born in the United States? **(SINGLE RESPONSE) (LOCK ORDER)**
11. Yes **(PROGRAMMER: ASSIGN 14 POINTS)**
12. No **(PROGRAMMER: ASSIGN 0 POINTS)**
13. **(ASK IF NOT BORN IN US, Q78=2)** At what age did you come to the US? **(NUMERIC OPEN END)**

_______ year(s) old **(PROGRAMMER: MINIMUM 1; DATA PROCESSOR: MAXIMUM MUST NOT EXCEED RESPONDENT’S AGE IN Q3)**

Under 1 year old

**(PROGRAMMER: ASSIGN 8 POINTS FOR UNDER AGE 10, 6 POINTS FOR AGE 10-17, 4 POINTS FOR AGE 18 TO 29 AND 0 POINTS FOR AGE 30+)**

1. **(ASK IF NOT BORN IN US, Q78=2)** How long have you been in the US? **(NUMERIC OPEN END)**

_______ year(s) **(PROGRAMMER: MINIMUM 1; DATA PROCESSOR: MAXIMUM MUST NOT EXCEED RESPONDENT’S AGE IN Q3)**

Less than 1 year

**(PROGRAMMER: ASSIGN 0 POINTS FOR LESS THAN 5 YEARS, 1 POINT FOR 5-9 YEARS, 2 POINTS FOR 10-14 YEARS, 3 POINTS FOR 15 TO 19 YEARS, 4 POINTS FOR 20 TO 29 YEARS, 5 POINTS FOR 30+ YEARS)**

1. **(ASK IF HISPANIC OR LATINO AND SPEAK SPANISH, Q5=3 & Q1=2)** Thinking about Latino and American cultures and lifestyles, would you say…? **(SINGLE RESPONSE) (LOCK ORDER)**
2. I prefer my Latino culture and language and usually avoid the American culture **(PROGRAMMER: ASSIGN 0 POINTS)**
3. I mostly prefer my Latino culture, but do include some parts of American culture **(PROGRAMMER: ASSIGN 3 POINTS)**
4. I like both Latino and American cultures and my lifestyle is equal parts of both **(PROGRAMMER: ASSIGN 6 POINTS)**
5. I mostly prefer the American culture, but do include some parts of Latino culture **(PROGRAMMER: ASSIGN 9 POINTS)**
6. I prefer my American culture and language and usually avoid the Latino culture **(PROGRAMMER: ASSIGN 12 POINTS)**

**AcculturationLevel. AUTOCODE: ACCULTURATION SCORING (AMONG RESPONDENTS WHO ARE HISPANIC OR LATINO AND SPEAK SPANISH)**

**ASSIGN ACCULTURATION SCORE BASED UPON ACCULURATION QUESTION INSTRUCTIONS**

**AUTOCODE: ACCULTURATION LEVEL (AMONG RESPONDENTS WHO ARE HISPANIC OR LATINO AND SPEAK SPANISH)**

1. **Un-acculturated (0 to 6 points)**
2. **Partially un-acculturated (7 to 13 points)**
3. **Bicultural (14 to 20 points)**
4. **Partially acculturated (21-27 points)**
5. **Acculturated (28-34 points)**
